# Assessment of Knowledge, Attitudes, and Propensity towards HPV Vaccine of Young Adult Students in Italy

**DOI:** 10.3390/vaccines8010074

**Published:** 2020-02-07

**Authors:** Cecilia Trucchi, Daniela Amicizia, Silvio Tafuri, Laura Sticchi, Paolo Durando, Claudio Costantino, Federica Varlese, Bruno Di Silverio, Anna Maria Bagnasco, Filippo Ansaldi, Giancarlo Icardi

**Affiliations:** 1 A.Li.Sa. Liguria Health Authority, 16121 Genoa, Italy; daniela.amicizia@unige.it (D.A.); federica.varlese@regione.liguria.it (F.V.); bruno.disilverio@regione.liguria.it (B.D.S.); filippo.ansaldi@unige.it (F.A.); 2 IRCCS San Martino Hospital, 16132 Genoa, Italy; sticchi@unige.it (L.S.); paolo.durando@unige.it (P.D.); icardi@unige.it (G.I.); 3 Department of Health Sciences, University of Genoa, 16132 Genoa, Italy; annamaria.bagnasco@unige.it; 4 Policlinico General Hospital, 70124 Bari, Italy; silvio.tafuri@uniba.it; 5 Department of Health Promotion Sciences, Maternal and Infant Care, Internal Medicine and Excellence Specialties (PROMISE), University of Palermo, 90127 Palermo, Italy; claudio.costantino01@unipa.it

**Keywords:** HPV, knowledge, practice, attitude, young adults, vaccination, student, prevention, sexually transmitted infection

## Abstract

Background: Human Papillomavirus (HPV) is a common sexually transmitted infection (STI), representing the main cause of genital warts and cervical cancer. This cross-sectional study evaluated knowledge and attitudes about HPV infection, related diseases, and prevention and propensity towards HPV vaccine among undergraduate students. Methods: An online and written survey about HPV and its prevention, targeted to young adults of both genders, was addressed to students attending health sciences and other schools at Universities of Genoa and Bari. Results: The overall median knowledge and attitude scores were 56.3% (25–75 *p* = 40–68.8%) and four out of five (25–75 *p* = 4–5), respectively. In the multivariate analysis, attending a health sciences university, using social networks ≤2 h a day, a history of STI, having heard about HPV and HPV vaccine previously resulted as predictors of higher knowledge scores. Having heard about HPV previously also predicted a high attitude score, together with a perceived economic status as good. Having Italian and healthcare worker parents, being employed, and following a specific diet, instead, predicted lower attitude score. Conclusions: Poor knowledge and good attitudes were found among undergraduates about HPV. In order to increase HPV vaccine compliance and the counselling skills of future healthcare workers, the improvement of training on HPV is needed.

## 1. Introduction

Human papillomavirus (HPV) infection is recognized as the most common STI worldwide [1,2]. HPV infection is acquired soon after sexual debut, it is transient and cleared at an early age [3]. If persistent, it can lead to a wide range of benign lesions and cancers, affecting the ano-genital and the oropharyngeal tract in both sexes [4]. Of note, official epidemiological estimates report 570,000 cases and 311,000 deaths due to cervical cancer in 2018 worldwide, placing the disease as the fourth most frequently diagnosed cancer and the fourth leading cause of cancer death in women [5]. The risk factors involved in the evolution of HPV-related diseases are the efficiency of the host’s immune response and the characteristics of the virus, including the genotype, the presence of multiple HPV infections, the viral load and integration in the host genome [6]. Co-factors related to the development of ano-genital cancers are alcohol, smoking, and the presence of co-infections with other pathogens [7,8].

Data acquired in recent years confirm that HPV vaccines and screening are the most effective prevention tools [9]; in particular, solid evidence is accumulating about vaccine effectiveness, long-lasting protection, and economic benefits in the avoiding cases [10,11]. Indeed vaccination programs have been implemented in 74 countries all over the world (33 countries in the WHO European Region) targeting girls and 11 targeted to boys, achieving successful results [12]. In Italy, the HPV vaccine started in 2007, with a strategy of active call and free-of-charge vaccines targeting girls aged 11–12 years [13]. Then the vaccination program was extended to males aged 11–12 years and at-risk subjects (men who engage in same-sex sexual behaviors) [14,15] following the latest evidence about the cost-effectiveness of HPV vaccination in other targets [16]. Furthermore, women aged 25 years, at the moment of cervical cancer screening can benefit from the HPV vaccine, as recommended by the National Vaccine Plan 2017–2019.

Despite this prevention opportunity, the coverage rate is still suboptimal in Italy, as in other countries, due to HPV vaccination uptake barriers, such as inadequate knowledge of HPV infection and related diseases, vaccination safety concerns, organizational aspects of HPV vaccine campaigns, and difficulties in vaccine schedule completion for subjects and providers [17]. In order to investigate the level of knowledge of HPV infection and vaccination, as well as the attitudes towards recommending the anti-HPV vaccine, an ad hoc knowledge and attitudes questionnaire was developed and administered to Italian young adult students.

## 2. Materials and Methods

### 2.1. Study Design and Participants

A cross-sectional multicenter survey about HPV and its prevention, targeted to young adults of both genders, was carried out from May 2017 to December 2018. The study population included females who retain the right to free-charge vaccination, males who can benefit from the vaccine in co-payment regimens, and subjects at risk, like those who are HIV-positive and men who have sex with men. Recruitment of the study participants took place at the University of Genoa (Liguria region, Northwest Italy), University of Bari (Apulia region, Southern Italy), and the San Martino Hospital in Genoa. In particular, subjects were recruited among students attending healthcare profession schools (i.e., nurses and healthcare assistants) who are required to periodically undergo a medical examination at occupational health surveillance outpatient clinics and who are attending academic schools including public health and prevention subjects in the first and second academic years. The study staff enrolled the students at the outpatient clinic and on the occasion of academic lessons. The sample size was calculated considering the number of subjects belonging to the target study population, the at-risk subjects, and healthcare personnel, the available evidence about the HPV vaccine acceptability fixing the confidence interval at 95%, a margin of error (+/−) of 2.5%, and adding a quota of 5%, in consideration of the possible drop-outs and missed answers.

### 2.2. Data Collection and Questionnaire

Ad hoc anonymous questionnaires addressed to young adults were developed. They were self-administered using the Google Drive platform that automatically populates and saves digital responses into a secure Microsoft Excel database protecting participant confidentiality throughout the surveying process. A consent page reporting the study aims and objectives, the participant’s right to the refusal or the possibility to terminate the participation in the study at any time and without disadvantages was created.

Participants could also fill two written informed consent and questionnaires characterized by the same structure of those administered online. The first questionnaire was set in two sections: the first one investigated the country of parent’s birth, level of parent’s education, parent’s occupation, and, for the participant, demographics, level of education, occupation, economic status, professed religion, healthy behavior, such as following a specific nutritional regimen (diet) and sport practice, sexual behaviors and orientation, use of the Internet and social networks, the sources used for seeking information on sexually transmitted diseases (STDs), including HPV, and remembering about vaccines received during childhood. The first section also included a 10-point Likert scale ranking the interest to receive an HPV vaccine. The second section included 13 questions on the knowledge on HPV infection, related diseases, and vaccine. The second questionnaire assessed their attitude using nine statements with a five-point Likert scale for each one (5 points- Strongly Agree, 4 point-Agree, 3 points-Neutral, 2 points-Disagree, 1 points-Strongly Disagree). It also included a 10-point Likert scale on how much the participant was interested to receive HPV vaccine after reading some information on HPV infection, related diseases, and vaccine included at the beginning of the second questionnaire (self-learning). Based on the participants’ responses, the knowledge, attitude, and intention scores were calculated. The ‘knowledge’ score summarizes the percentage of correct answers the participants gave among the knowledge questions. For each knowledge statement, one point was given for a correct answer (true or false) and zero points if they selected a wrong answer or “Do not know”. The total points were added for every participant and a percentage was calculated (number of correct answers/number of knowledge statements × 100). The higher the knowledge score, the more knowledgeable the participant is regarding HPV infection, its related diseases, and prevention. The attitude score was obtained by calculating the average of the participants’ responses to the statements on a five-point Likert scale. The median of the total points was calculated for every participant obtaining an attitude score ranging from 1.0 to 5.0. The closer the attitude score to 5, the more positive the participant’s attitudes. The “intention” score reflects the propensity toward the HPV vaccine. The “intention” score was estimated both at the beginning and at the end of the survey, when the information about HPV infection and vaccination had been given. The score is a scale from 1 to 10, where 10 reflects the highest propensity toward the HPV vaccine.

### 2.3. Statistical Analyses

Statistical analysis was performed by means of JMP software (SAS, NC), version 13 and Microsoft Excel, version 2016 after generating data from the written questionnaires and Google Drive (CA, USA). Median and 25–75 percentiles for continuous variables, frequencies, and percentages for categorical variables were calculated. The possible association of baseline characteristics of young adults with knowledge and attitude scores was tested through univariable logistic regression. A *p*-value less than 0.05 was considered significant throughout the study. Multivariate logistic regression analysis was performed to identify the predictors of having high knowledge score or positive attitude score towards HPV vaccination. Only covariates that showed a significance level of *p*-value < 0.1 in the univariate analysis were included in the multivariable regression model, using a backward stepwise algorithm. Given the high number of potential independent variables, a backward stepwise algorithm was used to identify the best-fitting subset of variables to use in the final regression model. In particular, Akaike’s Information Criterion (AIC) was used to assess the models’ fit and the model with the lowest AIC was selected for the multivariable analysis. β-coefficients (95% C.I.) and *p*-values were estimated.

### 2.4. Ethics Approval and Consent to Participate

In complying with the highest ethical standards, participants were informed that their participation was entirely voluntary and that they could withdraw at any moment. All data obtained through their participation was kept strictly confidential among the research team. In addition, an informed consent was obtained after explaining the nature of the study and its possible consequences of it. The study protocol was approved by the Liguria Regional Ethics Committee (P.R. 162REG2017) for the study coordination center.

## 3. Results

### 3.1. Participants’ Characteristics

A total of 680 young adults returned the self-administered questionnaires, giving a response rate of 100%. Table 1 shows the participants’ baseline characteristics. The median age of the participants was 20 (25–75 *p* = 19–21), 65.2% were female and 95.9% were Italian. A total of 97.1% declared to be students and only 2.9% were employed. A total of 24.5% of subjects had a graduate parent and 10.4% had at least one parent who was a health care operator. A total of 53.3% of the subjects consider their own economic status as good. As regards lifestyle habits, 23.1% and 56.6% of participants follow a specific diet and practice sports, respectively. Furthermore, 65% and 49.6% of young adults surfed the Internet and used the social networks up to 2 h a day, respectively. Among sexual habits, 61.8% of subjects had a partner at the time of the survey and 83.8% used contraceptive methods, the most reported of which was condom use (61.7% of cases). Furthermore, 97.2% of participants were heterosexual. The median age of sexual debut and number of partners in the last year were 17 (25–75 *p* = 16–18) and 1 (25–75 *p* = 1–1), respectively.

### 3.2. Sources of Information about HPV

A total of 80.4% of subjects had heard about HPV vaccine before the survey, in particular from a family member (43.7% of cases), Internet (24% of cases), teachers (25.1% of cases), and LHAs informative material (23.6% of cases) (Table 2).

### 3.3. Knowledge of HPV Infection, Related Diseases, and Prevention

The overall median knowledge score was 56.3% (25–75 *p* = 40–68.8).

Most of the subjects answered correctly the statement that HPV is sexually transmitted (82.4%) and that using condoms reduces the risk of HPV infection (86.2%) (Table 3). Only 40.3% knew that HPV infection can be transmitted during pregnancy and delivery from mother to the child. Among risk factors of exposure to HPV infection, only about 50% of participants recognized the early sexual debut. A total of 77.5% of the subjects knew that HPV infects both females and males, but only 21.4% and 54% correlates HPV to penile and cervical cancers, respectively. In terms of benign diseases, the information that HPV can cause genital warts is known by 42.8% of subjects, and 80.4% are aware that both females and males can develop them. Only 52.7% and 48.7% of subjects are aware that HPV infection is widespread and can be transmitted by asymptomatic carriers. Furthermore, 40.9% of participants think that most infected subjects develop cancer. As regards HPV therapy, only 23.4% of participants knew that no specific treatments are available. Finally, 67.8% and 58.8% of young adults correctly answered the questions related to the indication of HPV vaccine targeting both females and males and the efficacy of HPV vaccine in adults who already had a sexual debut.

Table 4 shows the results of univariable logistic regression investigating the possible association between the characteristics of the participant young adults and the knowledge score. Variables significantly related to higher knowledge scores are: attending a healthcare university (*p* < 0.0001), using social networks up to 2 h a day (*p* = 0.0215), history of sexual transmitted infection and of HPV-related lesions (*p* = 0.0349 and *p* = 0.0290, respectively), and having heard about HPV before the survey (*p* = 0.0035). Furthermore, family members and mass media as source of information about HPV are significantly related to worse knowledge scores (*p* = 0.0501 and *p* = 0.0762, respectively). Gynecologists, instead, are reported as a source of information by young adults with significantly higher knowledge scores (*p* = 0.0014).

### 3.4. Attitudes about HPV Infection and Prevention

The overall median attitude score was as high as 4 (25–75 *p* = 4–5). Only 49.7% of participants agree or strongly agree that during the own life he/she will have high probability to be exposed to HPV infections (Table 5). A total of 93.8% of subjects, instead, are aware that HPV can cause serious diseases. As regards the favorite source of information and opinion to get vaccinated, the most reported were trusted doctors (85.3%) and teachers (90.4%), followed by parents and partners (51.8% and 39.9%, respectively). About safety concern, 76.5% of partipants believe that HPV vaccine is safe. Furthermore, 83.5% agree or strongly agree to have their own child vaccinated.

Table 4 shows the results of univariable logistic regression investigating the possible association between the characteristics of the participant young adults and the attitude score. Significantly higher attitude scores were reported among subjects with foreign parents, without parents who are healthcare workers (*p* = 0.0103), who are not employed (*p* = 0.0211), who reported a good economic status (*p* = 0.0031), who do not follow a specific diet (*p* = 0.0004), who use social network up to 2 h a day (*p* = 0.0115), those without immunodeficiencies (*p* = 0.0381), and those who heard about HPV before the survey (*p* < 0.0001). As regards the source of information about HPV vaccine, subjects who reported teachers, LHAs informative material, pediatricians/general practitioners, had significantly higher attitude scores (*p* = 0.0100, *p* = 0.0081, and *p* = 0.0061, respectively).

### 3.5. Propensity toward HPV Vaccination

The median propensity score before and after the educational intervention was stable and high as 9 (25–75 *p* = 8–10).

### 3.6. Multivariate Analysis

The results of the multivariate analysis predicting high knowledge and attitude scores are presented in Table 6 and Table 7, respectively. The predictors of high knowledge are attending Healthcare University, using social network ≤2 h/day, having a history of STIs, and having heard about HPV and anti-HPV vaccine before the survey. As regards predictors of high attitude score, a good economic status and having heard about HPV before the survey are predicted to have more positive attitudes towards HPV vaccine, while having Italian parents, being employed, and a parent who is/was a healthcare worker are predicted to have worse attitudes. Both the models significantly help in predicting knowledge and attitude scores since the *p*-values of the *F*-test are <0.0001.

## 4. Discussion

To the best of our knowledge, this is one of the first studies to quantitatively investigate knowledge and attitudes about HPV, and propensity towards HPV vaccination before and after a formative moment, in young adults students of both genders living in a high gross domestic product (GDP) country such as Italy, where a dynamicity in the objectives and targets of HPV vaccination by public health since its introduction in 2007 was observed. This study was performed not only in undergraduates attending health sciences schools, but also in other university students.

Young adults are a category at risk of sexually transmitted HPV infection. Since HPV has a causative role in serious and frequent diseases, such as cancers and genital warts, respectively, interventions to prevent low and high-risk types of HPV infections are urgently warranted; indeed, vaccinating both men and women against HPV is crucial to protect against HPV-related cancers and other diseases.

Recent international recommendation on HPV vaccination have included additional age classes, not only adolescents of both genders (aged 11–12 years), but also females 13–26 years-old who have not been previously vaccinated or who have not completed the vaccine series, and males aged 13–21 years and 22–26 years with special conditions or who want to protect themselves from the disease [17,18]. The current Italian National Vaccine Prevention Plan (NVPP) 2017–2019 extended the vaccination program to include pre-adolescent males (aged between 11 and 12 years) and at-risk subjects (men who engage in same-sex sexual behaviors) [14]. Furthermore, the NVPP recommends a multi-cohort immunization strategy, preferably targeting 25 years-old young adult females who begin cervical cancer screening. Based on the current vaccination plans and the need to implement knowledge and attitude of young adults of both gender, an overall approach is desired. Indeed, it is of note that HPV coverage rates are suboptimal in both genders in many developed countries, including Italy [19].

An evaluation of what young adults know about the threat of HPV and believe about HPV prevention can offer possible solutions to remedy the underutilization of the HPV vaccine in a population at high risk for contracting this preventable infection and to explore innovative strategies. The findings are, therefore, relevant at the light of better understanding and comparing educational needs and attitudes toward HPV infection and prevention in one of the HPV vaccine targets [20,21,22]. Furthermore, the interviewed subset including health sciences students, allowed estimating knowledge needs and main attitudes of future healthcare workers and counsellors.

Our findings highlight, on one side, insufficient knowledge of undergraduate students on HPV infection and HPV- related diseases, on the other side, a substantial positive attitude toward the vaccine. Gaps on HPV knowledge were detected in previous studies enrolling study population similar to our study population for demographic characteristics [23,24,25,26]. In our survey, a not negligible percentage of students had low consciousness about the impact of HPV infection in terms of spread, the routes of infection, and risk factors, such as early sexual debut, the role of HPV in causing penile cancer and genital warts, and the unavailability of specific therapeutic treatment. This would confirm the need to promote educational programs for young subjects and introducing changes in the current curriculum of university students attending health sciences schools, in order to increase understanding about HPV of future healthcare professionals. However, as expected, higher knowledge was detected in health sciences students’ respect to undergraduates attending other academic course (*p* < 0.0001). Results worthy of highlighting are that more than 93% affirmed that HPV can be implicated in developing serious disease but only 49.7% consider themselves at risk of exposure to HPV. These results are comparable with those described by Villanueva et al. who recently investigated knowledge, attitudes, and intentions towards HPV among nursing students in Spain [25]. As regards attitudes towards HPV vaccine, more than 90% and 76% of participants consider HPV vaccine effective and safe, respectively. In a very recent Italian cross-sectional study that assessed nursing students’ knowledge and attitudes about HPV infection and vaccination, only 20.5% of subjects considered very safe the HPV vaccine [27]. The abovementioned Spanish study also investigated attitudes towards HPV vaccine efficacy and safety, finding that 66.5% and 65.4% of students agree or strongly agree that HPV is capable of preventing the occurrence of cervical cancer and that side effects are reasonable, respectively [25]. We also investigated the willingness of participants considered as potential future parents to get their own child immunized against HPV and only 83.5% agree or strongly agree with this sentence. This observation is of concern since it demonstrates that the high consciousness of the causal role of HPV in determining serious diseases, such as cancers, is not a sufficient factor in determining recommended vaccine compliance.

Among young adults characteristics, parents’ nationality, educational qualification, and employment were investigated in relation to knowledge and attitude scores. The knowledge score resulted higher, although not statistically significant, in young adults with healthcare worker parents, while the attitude score was higher for subjects with parents who are employed in other sectors. This observation could be linked to the well-known largely suboptimal compliance of healthcare workers to vaccines [28,29]. Furthermore, considering the parents’ nationality, knowledge scores of young subjects was similar between those with Italian parents and those without. On the contrary, the attitude score was higher in those with foreigner parents (*p* = 0.0491), who mainly come from developing countries. A possible explanation could be a better propensity by the foreign families towards Italian healthcare services, including prevention. As regards lifestyles, the young who follow specific nutritional regimen had significantly lower attitude score (*p* = 0.0044), confirmed by multivariate analysis (*p* < 0.0001), this could be explained by the fact that those who engage in specific behaviors oriented around ‘the natural’ is often hesitant or refuse vaccines [30]. Surprisingly, physical activity was related neither to higher knowledge nor to attitude scores. Although healthy lifestyles are expected to be related to better compliance to preventive measures. Among sexual habits, 83.8% of subjects who had a partner at the time of the survey (61.8% of participating young adults) used contraceptive methods, the most represented of which was condom use (61.7%), followed by oral contraceptive (31.8%). Furthermore, the median age of sexual debut was 17 (25–75 *p* = 16–18), with a median of lifetime sexual partners of 2 (25–75 *p* = 1–4). These findings are substantially comparable with the observations obtained in other European countries [22,25]. Considering sexual orientation, 1.3% and 1.5% were bisexual and homosexual, respectively. Of relevance, homosexual surveyed subjects demonstrated higher knowledge than heterosexual and bisexual subjects, although this result has to be interpreted with caution because of the scarce number of surveyed homosexual and bisexual subjects. A possible explanation could be the higher attention toward to health and prevention tools by homosexuals. History of STIs and HPV-related lesions was also investigated, showing that only 4.9% and 2.2% of subjects were diagnosed for the abovementioned diseases, respectively. Furthermore, the history of STIs was found as predictor of high knowledge score at the multivariate analysis (*p* = 0.0440). This observation could be linked to the previous contact of affected subjects with the healthcare system which could have favored the acquisition of knowledge on this topic.

Before the educational moment, subjects were asked to declare if they have heard about HPV previously. Our findings revealed that a high percentage of young (80%) already knew this infection, which is well up rates reported in other published research [31,32,33] and significantly predicts both higher knowledge and attitude scores (*p* = 0.0072 and *p* < 0.001). In particular, family members, Internet, teachers, and LHAs’ informative material were the most frequently reported sources of information. Surfing the web and the use of social networks were also investigated, showing that using social network ≤2 h/day is among the predictors of better knowledge on HPV compared to higher use (*p* = 0.0141). This finding suggests that social networks can also disseminate misleading messages on HPV. Nevertheless, the increasing pervasiveness of social media, such as Facebook, as powerful communication channels means that they can potentially be used to effectively reach young people. Therefore, public health should better communicate messages through existing networks and engage in on-going dialogue with users [34,35,36].

Our study also deepened attitudes regarding the favorite sources of information about HPV of participating subjects, finding that talking about HPV with teachers and trusted doctor is considered very useful by young adults, while only 51.8% and 39.9% of participants believe that parents’ and partner’s opinions are essential to decide to get vaccinated against HPV. These answers confirm the role of physicians and teachers recognized by young adults in spreading health information including sensitive topics such as STIs. Of note, the majority of participants (83.92%) agree or strongly agree to make compulsory HPV vaccine for boys and girls before the sexual debut and for at risk subjects. This is of interest in countries such as Italy where a recent law introduced the compulsory of some vaccines, excluding HPV [37].

In order to evaluate the efficacy of educational intervention in implementing the propensity of young adults toward HPV vaccine, we estimated it through a score before and after the administration of a written text summarizing the available evidences on HPV infection, related diseases and prevention. The score resulted very high (median = 9, 25–75 *p* = 8–10) and remained stable after the educational session. A recent meta-analysis that assessed the effectiveness of interventions targeting HPV vaccine initiation and completion among children, adolescents, and young adults aged 9–26 years support behavioral and informational interventions, including education (e.g., exercises, audit and feedback, video intervention, peer/medical narrative), educational websites tailored to baseline knowledge, and brochures/factsheets, for HPV vaccine initiation and behavioral interventions for completion [38]. Considering the surveyed subset of health sciences students, and their future role in counselling on prevention themes, a comparison with the results of a recent published study by Berenson et al. could be conducted. The authors assessed the effects of an educational program by means of a single lecture and delivered by an expert and found that this can improve medical students’ attitudes and comfort with HPV vaccine counseling [39]. This evidence suggests that short educational interventions are already efficacious, but the presence of an expert open to discussion allows obtaining better results than reading informative material alone.

The study limits are the nature of self-reported data collected from the participants, which may be compromised by the respondents’ memory of experiences of interest. Furthermore, since northern and southern regions were involved in the enrollments, the representativeness of young adults living in the Central Italian regions is lacking. Despite these limitations, this survey obtained a very high response rate, demonstrating the strong interest in HPV topic. As regards education, the self-learning session could be improved by focus groups and other interactive methods. Nevertheless, academic public health teaching includes HPV burden and prevention among treated themes.

## 5. Conclusions

The results of the current study demonstrates that Italian undergraduates show suboptimal knowledge on HPV and good attitudes. Since they represent both the target of HPV vaccine and future healthcare workers, the implementation of training about HPV infection, its related diseases, and prevention tools are needed to support the important role they play in the prevention of STIs, some of which are related to the occurrence of cancer. Universities represent a privileged setting to spread information about HPV to the target audience and young adults recognize teachers together with trusted doctors as the favorite source of information. Improving students’ knowledge may have favorable implications in increasing vaccine coverage rates.

## Figures and Tables

**Table 1:** Baseline characteristics of the study’s participants.

	*N (%)*	*Median (25–75 p)*
**Total**	680 (100)	
**Variables**		
**Age**		20 (19–21)
**Sex**		
Female	443 (65.15)	
Male	237 (34.85)	
**Birthplace**		
Italy	652 (95.88)	
NR	2 (0.29)	
**Parents’ nationality**		
Italian parents	515 (75.74)	
NR	146 (21.47)	
**Educational qualification of parents**		
Primary School Certificate	2 (0.39)	
Secondary School Certificate	153 (29.94)	
High School Diploma	231 (45.21)	
University Degree	125 (24.46)	
NR	169 (24.85)	
**Parents employed**	507 (74.56)	
NR	159 (23.38)	
**Parents healthcare worker**	50 (10.35)	
**Student**	660 (97.06)	
NR	20 (2.94%)	
**University students**	597 (90.45)	
Health sciences	186 (31.26)	
Other	393 (65.85)	
NR	18 (2.87)	
**Worker**	147 (21.62)	
**Economic status**		
Acceptable	239 (35.15)	
Good	364 (53.53)	
Poor	77 (11.32)	
**Diet**	157 (23.09)	
**Physical activity**	385 (56.62)	
**Surf the web**		
≤2 h	436 (64.98)	
3–4 h	184 (27.42)	
≥5 h	51 (7.6)	
**Use of social network**		
≤2 h	330 (49.55)	
3–4 h	233 (34.98)	
≥5 h	103 (15.47)	
**Religion**	449 (66.03)	
Catholics	425 (94.65)	
**Cohabitants**		
Parents	580 (85.29)	
Friends	60 (8.82)	
Alone	28 (4.12)	
Sexual Partner	12 (1.76)	
**Sexual Partner**	420 (61.76)	
**Use of contraceptive methods**	352 (83.81)	
**Type of contraceptive method**		
Condom	217 (61.65)	
Oral contraceptive	112 (31.82)	
Vaginal ring	25 (7.10)	
Intrauterine spiral	4 (1.14)	
**Sexual orientation**		
Heterosexual	661 (97.21)	
Bisexual	9 (1.32)	
Homosexual	10 (1.47)	
**Sexual Partners in the last year**		1 (1–1)
**Age of first sexual intercourse**		17 (16–18)
**Lifetime sexual partners**		2 (1–4)
**History of immunodeficiencies**	18 (2.65)	
**History of STIs**	33 (4.85)	
**History of HPV related lesions**	15 (2.21)	

Frequencies and percentages for categorical variables and median and 25–75 percentiles for continuous variables, were calculated. Abbreviations: Non-responded, NR; sexually transmitted infections, STIs.

**Table 2:** Sources of information about HPV.

Variables	*N (%)*
Ever heard about HPV	578 (85.00)
Ever heard about HPV vaccine	547 (80.44)
Family member	239 (43.69)
Internet	131 (23.95)
Teacher	137 (25.05)
Information from the Local Health Agency	129 (23.58)
Healthcare workers of a vaccine center	69 (12.61)
Pediatrician/General Practitioner	101 (18.46)
Radio/TV	89 (16.27)
Gynecologist	78 (14.26)
Newspapers	68 (12.43)
Friends	59 (10.79)
Nurse	27 (4.94)
Other specialist doctor	21 (3.84)
Other Healthcare professionals	21 (3.84)
Sexual Partner	16 (2.93)
Colleagues	10 (1.83)
Pharmacist	6 (1.10)
**Favourite source of information on HPV vaccines**	
Trusted doctor	387 (56.91)
Internet	330 (48.53)
Books/paper material	105 (15.44)
Friends	24 (3.53)

Frequencies and percentages for categorical variables and median and 25–75 percentiles for continuous variables, were calculated.

**Table 3:** Study’s participants’ knowledge about HPV infection, related diseases, and prevention.

Knowledge Statement	Response	Correct		Do not Know		Wrong	
		*N*	*%*	*N*	*%*	*N*	*%*
HPV infection is transmitted through sexual intercourse	True	560	82.35	95	13.97	25	3.68
If yes, early sexual debut increases the risk of contracting HPV	True	263	46.96	127	22.68	170	30.36
If yes, using condoms reduces the risk of HPV transmission	True	481	86.20	56	9.7	23	4.12
If yes, a high number of sexual partners increases the risk of contracting HPV	True	383	68.39	97	17.33	80	14.28
HPV infection can be transmitted from mother to child during pregnancy and during delivery	True	274	40.29	292	42.94	114	16.77
HPV can infect both males and females	True	527	77.50	−	−	153	22.50
Disease associated with HPV in men: penile cancer	True	114	21.39	221	41.46	198	37.14
Disease associated with HPV in women: cervical cancer	True	367	53.97	172	25.29	141	20.74
HPV can lead to genital warts	True	291	42.79	334	49.12	55	8.1
If yes, genital warts affect both male and female	True	234	80.41	−	−	57	19.58
HPV infection is widespread	True	358	52.65	246	36.18	76	11.17
Most HPV infected people develop cancer	False	118	17.35	278	40.88	284	41.76
HPV infection can be transmitted from a carrier to his/her partner only if the carrier shows symptoms	False	331	48.67	207	30.44	142	20.88
A person may be HPV-infected and not be aware of it	True	452	66.47	180	26.47	48	7.06
There are no specific therapies for HPV infection	True	159	23.38	319	46.91	202	29.71
Both males and females can be vaccinated against HPV	True	461	67.79	3	0.44	216	31.76
The HPV vaccine is also effective in after starting sexual activity	True	400	58.82	199	29.27	81	11.91

For each knowledge statement, the correct answer is reported. Frequencies and percentages of correct, wrong and unknown answers were calculated.

**Table 4:** Participants’ knowledge and attitude scores stratified by their characteristics.

Characteristics	Knowledge Score	*p-Value*	Attitude Score	*p-Value*
	*Median (25–75 p)*		*Median (25–75 p)*	
**Sex**		0.7976		0.0053
Female	56.3 (40–68.8)		4.5 (4–5)	
Male	53.3 (38.5–68.8)		4 (4–5)	
**Birthplace**		0.0716		0.7724
Italy	56.3 (40–68.8)		4 (4–5)	
Other Countries	48.35 (32.8–64.08)		4.5 (4–5)	
**Parents’ nationality**				0.0492
Italian	53.3 (37.5–64.7)	0.5907	4 (4–5)	
No	50 (33.3–62.5)		4.5 (4–5)	
**Educational qualification of parents**		0.6626		0.6358
Primary School Certificate	44.85 (25–64.7)		4.5 (4–5)	
Secondary School Certificate	52.9 (38–62.5)		4 (4–5)	
High School Diploma	53.3 (38.5–66.7)		4 (4–5)	
University Degree	56.3 (40–68.8)		4 (4–5)	
**Parents employed**	53.3 (37.5–64.7)	0.9090	4 (4–5)	0.3643
No	50 (42.85–63.05)		4 (4–4.13)	
**Parents healthcare worker**	58.15 (35.1–73.73)	0.1899	4 (4–4.5)	0.0103
No	53.3 (37.5–62.5)		4 (4–5)	
**University students**		<0.0001		0.2365
Health sciences	62.5 (50–80)		4.5 (4–5)	
Other	46.7 (34.3–60)		4.5 (4–5)	
**Worker**	53.3 (40–68.8)	0.7846	4 (4–5)	0.0211
No	56.3 (40–68.8)		4.5 (4–5)	
**Economic status**		0.3755		
Acceptable	53.3 (38.5–68.8)		4 (4–5)	0.0031
Good	56.3 (40–68.8)		4.5 (4–5)	
Poor	56.3 (37.5–62.5)		4 (4–4.5)	
**Diet**	53.3 (38.75–66.7)	0.6699	4 (4–4.75)	0.0004
No	56.3 (40–68.8)		4.5 (4–5)	
**Physical activity**	56.3 (40–68.8)	0.3145	4 (4–5)	0.0577
No	56.3 (39.63–68.8)		4.5 (4–5)	
**Surf the web**		0.9326		0.4214
≤2 h	56.3 (40–68.8)		4 (4–5)	
3–4 h	56.3 (40–68.8)		4 (4–5)	
≥5 h	53.3 (40–64.3)		4 (4–5)	
**Use of social network**		0.0215		0.0115
≤2 h	56.3 (42.9–68.8)		4.5 (4–5)	
3–4 h	53.3 (38.5–68.8)		4 (4–5)	
≥5 h	53.3 (33.3–62.5)		4 (4–4.5)	
**Religion**	53.3 (40–68.8)	0.4945	4 (4–5)	0.9570
No	56.3 (37.5–68.8)		4 (4–5)	
**Cohabitants**		0.5603		0.4981
Parents	56.3 (40–66.7)		4 (4–5)	
Friends	53.1 (31.8–76.13)		4.5 (4–5)	
Alone	64.6 (32.85–82.4)		4.5 (4–5)	
Sexual Partner	52.75 (33.3–64.15)		4 (4–4.5)	
**Sexual Partner**	56.3 (40–68.8)	0.5538	4 (4–5)	0.8926
No	56.3 (40–68.8)		4 (4–5)	
**Contraceptive methods used**	53.55 (40–66.7)	0.8904	4 (4–5)	0.5055
No	56.3 (35.7–73.3)		4 (4–5)	
**Sexual orientation**		0.3461		0.0841
Heterosexual	56.3(40–68.8)		4 (4–5)	
Bisexual	52.9 (36.5–59.4)		5 (4–5)	
Homosexual	72.8 (35.95–88.2)		4.75 (4–5)	
**Sexual Partners in the last year**		0.5801		0.1398
0–2	56.3 (40–68.8)		4 (4–5)	
≥ 3	52.9 (38.75–69.7)		5 (4–5)	
**Age of first sexual intercourse**		0.4739		0.4767
13–14	54.8 (35.1–65.2)		4.5 (4–5)	
15–17	53.3 (38.25–68.8)		4 (4–5)	
≥ 18	56.3 (41.7–68.8)		4 (4–5)	
**Lifetime sexual partners**		0.1491		0.1929
0–3	56.3 (38.5–68.28)		4 (4–5)	
≥ 4	56.3 (40–68.8)		4.5 (4–5)	
**History of immunodeficiency**	56.3 (38.5–63.05)	0.7165	4 (3.88–4.13)	0.0381
No	56.3 (40–68.8)		4 (4–5)	
**History of STIs**	62.5 (41.7–78.9)	0.0349	4.5 (4–5)	0.1241
No	56.3 (40–68.8)		4 (4–5)	
**History of HPV related lesions**	68.8 (46.2–88.2)	0.0290	4.5 (4–5)	0.4276
No	56.3 (40–68.8)		4 (4–5)	
**Ever heard about HPV**	56.3 (40–68.8)	0.0035	4.5 (4–5)	<0.0001
No	46.7 (33.3–62.95)		4 (3.5–4.3)	
**Ever heard about HPV vaccine**	56.3 (41.7–68.8)	0.0012	4.5 (4–5)	<0.0001
No	50 (34.5–62.5)		4 (3.75–4.5)	
**Source of information on HPV vaccine**				
Family member	56.3 (40–66.7)	0.0501	4.5 (4–5)	0.6436
No	57.95 (42.9–73.3)		4.5 (4–5)	
Internet	58.8 (42.9–73.3)	0.2843	4.5 (4–5)	0.8713
No	56.3 (40.43–68.8)		4.5 (4–5)	
Teacher	60 (43.8–72.8)	0.0773	4.5 (4–5)	0.0100
No	56.3 (40–68.8)		4.5 (4–5)	
Information from the Local Health Agency	58.8 (46.2–70.6)	0.1671	4.5 (4–5)	0.0081
No	56.3 (40–68.8)		4.5 (4–5)	
Healthcare workers of a vaccine center	56.3 (43.8–62.5)	0.2754	4.5 (4–5)	0.6951
No	56.3 (41.27–70.6)		4.5 (4–5)	
Pediatrician/General Practitioner	56.3 (43.8–68.8)	0.6522	5 (4–5)	0.0061
No	56.3 (40–68.8)		4.5 (4–5)	
Radio/TV	53.8 (33.3–65.7)	0.0762	4.5 (4–5)	0.4692
No	56.3 (43.57–70.6)		4.5 (4–5)	
Gynecologist	62.5 (46.7–81.57)	0.0014	4.5 (4–5)	0.0564
No	56.3 (40–68.8)		4.5 (4–5)	
Newspapers	56.3 (35.4–70.15)	0.7765	4.5 (4–5)	0.3178
No	56.3 (42.9–68.8)		4.5 (4–5)	
Friends	57.1 (43.8–66.7)	0.5906	4.5 (4–5)	0.4726
No	56.3 (40–68.8)		4.5 (4–5)	

For each knowledge statement, one point was given for a correct answer (true or false) and zero points if a wrong answer or “Do not know” was reported. The knowledge score is the number of correct answers/number of knowledge statements × 100. Attitude score of each subject was estimated through a five-point Likert scale (5 points—Strongly Agree, 4 point-Agree, 3 points-Neutral, 2 points-Disagree, 1 points-Strongly Disagree) and is the median of the total points was calculated for every participant (range =1.0 to 5.0). The univariable logistic regression tested the possible association of baseline characteristics of young adults with knowledge and attitude scores. A *p*-value less than 0.05 was considered significant.

**Table 5:** Study’s participants’ attitudes toward HPV infections, related diseases, and prevention statements.

Attitude Statement	Strongly Agree		Agree		Neutral		Disagree		Strongly Disagree	
	*n*	*%*	*n*	*%*	*n*	*%*	*n*	*%*	*n*	*%*
I believe that Papillomavirus (HPV) can cause serious diseases	380	55.88	258	37.94	29	4.26	8	1.17	5	0.74
I believe that I will be exposed to HPV infection during my life	77	11.32	261	38.38	225	33.09	89	13.09	28	4.12
I believe anti-HPV vaccines are capable of preventing the occurrence of cervical cancer and genital warts	338	49.70	280	41.18	56	8.24	6	0.88	−	
I believe the anti-HPV vaccination must be mandatory for boys and girls before the sexual debut and for at risk subjects	332	48.82	238	35.10	84	12.39	16	2.36	10	1.48
I believe my parents’ favorable opinion is essential to decide to get vaccinated against HPV *	114	18.57	204	33.22	169	27.52	83	13.52	44	7.16
I believe that the favorable opinion of my partner is essential to decide to get vaccinated against HPV	77	11.32	194	28.53	175	25.73	128	18.82	106	15.59
I believe it is useful to talk to my doctor about HPV infections and other sexually transmitted infections	243	35.73	337	49.55	87	12.79	8	1.18	5	0.73
I believe it is useful to talk about HPV infections and other sexually transmitted infections at school	364	53.53	251	36.91	58	8.53	5	0.73	2	0.29
I believe if I have children I get them vaccinated against HPV	324	47.65	244	35.88	102	15	10	1.47	−	
I believe the anti-HPV vaccine is safe	218	32.1	302	44.41	139	20.44	17	2.5	4	0.59

Legend: * N. 66 (9.71%) subjects did not answer. For each attitude statement, the answer is reported. Frequencies and percentages of “strongly agree”, “agree”, “neutral”, “disagree”, “strongly disagree” answers were calculated.

**Table 6:** Multivariate analysis for predicting knowledge score.

Characteristics	β-Coefficient (95% C.I.)	95% C.I. Lower Limit	95% C.I. Upper Limit	*p*-Value
University: health sciences	6.713	4.508	8.918	<0.0001
Use of social network ≤2 h/day	2.691	0.547	4.834	0.0141
History of STIs	4.208	0.114	8.302	0.0440
Ever heard about HPV	8.789	2.397	15.181	0.0072
Ever heard about HPV vaccine	6.061	1.262	10.859	0.0135

Covariates significant at 0.1 in the univariate analysis were evaluated for inclusion in multivariable regression model, using a backward stepwise algorithm.

**Table 7:** Multivariate analysis for predicting attitude score.

Characteristics	β-Coefficient (95% C.I.)	95% C.I. Lower Limit	95% C.I. Upper Limit	*p*-Value
Italian parents	−0.1548	−0.3031	−0.0065	0.0408
Worker	−0.0911	−0.1610	−0.0213	0.0106
Economic status: good	0.0713	0.0178	0.1246	0.0089
Diet	−0.1473	−0.2126	−0.0821	<0.0001
Ever heard about HPV	0.1715	0.1018	0.2413	<0.0001
Parents healthcare worker	−0.1132	−0.2001	−0.0263	0.0108

Covariates that were significant at 0.1 in the univariate analysis were evaluated for inclusion in multivariable regression model, using a backward stepwise algorithm.
